# Cuproptosis-related lncRNA signatures predict prognosis and immune relevance of kidney renal papillary cell carcinoma

**DOI:** 10.3389/fphar.2022.1103986

**Published:** 2022-12-21

**Authors:** Tongjin Xie, Bin Liu, Dongbo Liu, Yusong Zhou, Qingping Yang, Dai Wang, Mengjie Tang, Wei Liu

**Affiliations:** ^1^ Department of Urology, The Third Xiangya Hospital, Central South University, Changsha, China; ^2^ Department of Pharmacy, Zunyi Medical University, Zunyi, China; ^3^ Department of Pharmacy, The Third Xiangya Hospital, Central South University, Changsha, China; ^4^ Xiangya School of Pharmacy, Central South University, Changsha, China; ^5^ Department of Pathology, Hunan Cancer Hospital, The Affiliated Cancer Hospital of Xiangya School of Medicine, Central South University, Changsha, China

**Keywords:** lncRNA, cuproptosis, KIRP, prognosis, immune relevance

## Abstract

Kidney renal papillary cell carcinoma (KIRP) has a high mortality rate and a poor prognosis. Cu concentrations differed significantly between renal cancer tissues and adjacent normal tissues. Cuproptosis is a newly identified cell death. Long non-coding RNAs (lncRNAs) play a crucial role in the progression of KIRP. In this study, we focused on constructing and validating cuproptosis-related lncRNA signatures to predict the prognosis of KIRP patients and their immune correlation. We created prognosis models using Cox regression analysis and the least absolute shrinkage and selection operator (LASSO) algorithm. We found that patients in the high-risk group had poorer overall survival (OS) and progression-free survival (PFS) and higher mortality. Risk score and stage are prognosis factors independent of other clinical features. Kaplan-Meier analysis, receiver operating characteristic (ROC) curves, and C-index curves showed that cuproptosis-related lncRNA signatures could more accurately predict the prognosis of patients. Functional enrichment analysis suggests that the function of differentially expressed genes (DEGs) is associated with KIRP development and immunity. In immune-related function analysis, we found a significant difference in parainflammation responses between high-risk and low-risk groups. The mutation frequencies of TTN, MET, KMT2C, PKHD1, SETD2, and KMT2D genes in the high-risk group were higher than those in the low-risk group, but the mutation frequencies of MUC16, KIAA109, CUBN, USH2A, DNAH8 and HERC2 genes were significantly lower than those in the low-risk group. Survival analysis of tumor mutation burden (TMB) and combined TMB-risk showed better OS in patients with high TMB. Immune infiltration and immune checkpoint analysis assessed the immune association of six high mutation frequency genes (TTN, MET, KMT2C, PKHD1, SETD2, and KMT2D) with KIRP. Finally, we performed a drug sensitivity analysis and screened 15 potential drugs that differed between high-risk and low-risk patients. In this study, we constructed and validated cuproptosis-related lncRNA signatures that can more accurately predict the prognosis of KIRP patients and provide new potential therapeutic targets and prognosis markers for KIRP patients.

## Introduction

Kidney renal papillary cell carcinoma is the second type of renal cancer incidence, accounting for approximately 15%–20% of renal cell carcinoma (Hong et al., 2021). The treatment modalities of KIRP include traditional surgical treatment, molecular targeted therapy, and chemoradiotherapy, while molecular targeted therapy is an important treatment option to improve the quality of life of KIRP patients. However, KIRP-related therapeutic targets and prognosis markers are very lacking. Therefore, finding new therapeutic targets and prognosis markers for KIRP has important clinical implications.

LncRNAs are RNA transcripts > 200 nucleotides in length (Wang et al., 2019) that are widely involved in vital physiological processes such as metabolism and immunity and are closely related to the development of diseases such as tumors, cardiovascular diseases, neurological diseases, and nephropathy. Some studies have shown that lncRNA expression is significantly associated with the diagnosis and prognosis of KIRP (Lan et al., 2017; Zuo et al., 2018; Kang and Yang, 2022; Wu et al., 2022). Copper is essential during metabolism, including iron uptake and mitochondrial respiration (Ruiz et al., 2021). Increasing evidence suggests that copper is involved in multiple processes of tumor growth (Oliveri, 2022) and plays a vital role in the development of tumors. Excess copper may lead to mitochondrial protein aggregation and show different forms of cell death (Kahlson and Dixon, 2022). Cuproptosis is a novel programmed cell death discovered by Tsvetkov et al. (2022). Recently, some studies have found that cuproptosis-related lncRNAs are involved in the development of kidney renal clear cell carcinoma (KIRC), bladder cancer, and colorectal cancer (Cancer Genome Atlas Research Network, 2014; Kim et al., 2022; Zhu et al., 2022). However, cuproptosis-related lncRNA-related studies are currently lacking in KIRP.

Therefore, it is essential to identify the prognosis signatures and associated underlying mechanisms of cuproptosis-related lncRNAs in KIRP. In this study, we found for the first time that cuproptosis-related lncRNA signatures can predict the prognosis and immune correlation of KIRP and provide new potential therapeutic targets and prognosis markers for KIRP patients.

## Materials and methods

### Data processing and identification of cuproptosis-related lncRNAs

We obtained 290 KIRP patients from the Cancer Genome Atlas (TCGA) database; see Supplementary Table S1 for clinical details. In the Cancer Genome Atlas–Kidney renal papillary cell carcinoma database (TCGA-KIRP, https://portal.gdc.cancer.gov/), we obtained 321 RNA sequencing data, 291 clinical datasets, and 282 gene mutation data for KIRP patients. Data analysis was performed using R (version 4.2.1) and R Bioconductor packets. We used the “limma” software package to perform co-expression correlation analysis between cuproptosis-related gene expression profiles and lncRNAs to identify cuproptosis-related lncRNAs.

### Construction of the prognosis cuproptosis-related lncRNA signature

From previous studies (Aubert et al., 2020; Bian et al., 2022; Chen, 2022), we obtained 19 cuproptosis-related genes (Supplementary Table S2). TCGA-KIRP data were randomly divided into training and testing groups at a ratio of 1:1. Subsequently, we performed Lasso regression analysis to identify cuproptosis-related lncRNAs. Univariate Cox regression analysis (*p* < 0.05) was performed in the training group to determine whether these lncRNAs were associated with patient prognosis in the training group. Multivariate regression analysis (*p* < 0.05) identified 11 cuproptosis-related lncRNAs as independent prognosis factors. Then, using the best model parameters, construct risk signatures and calculate risk scores. Resulting model risk score = explncRNA1*coef-lncRNA1 + explncRNA*coef-lncRNA2 + … + explncRNAi*coef-lncRNAi.

### Survival analysis of the signature

To validate the prognosis power of the model, we divided the KIRP sample into high-risk and low-risk groups based on the median risk score. Using the survival package, we analyzed OS and PFS in different risk groups of KIRP patients. We performed univariate and multivariate regression Cox analyses to assess the prognosis value of the risk signatures. The R package “pheatmap” was used to visualize clinicopathological variables in the high-risk and low-risk groups from the entire set of TCGA-KIRP samples and to draw a heatmap of patient survival status and lncRNA expression.

### Independent analysis of the prognosis factor

To determine whether these risk signatures could be independent prognosis factors, we performed univariate and multivariate Cox regression analyses using the “survival” package. We used the “survminer” and “timeROC” packages to calculate the 1-year, 3-year, and 5-year area under the ROC curve (AUC) of the risk signature in training, testing, and all groups.

### Building nomogram and principal component analysis

Using the R software packages “rms” and “regplot,” we constructed a nomogram to predict the survival of KIRP patients at 1, 3, and 5 years. Calibration curves were used to assess whether predicted survival was consistent with actual survival. We randomly selected one patient to confirm the predictive utility of the nomogram. We used c-index curves to validate the reliability of the prognosis model. Finally, to determine whether these lncRNA signatures could predict KIRP patients at different stages, we divided patients into stages I-II and III-IV. Using “limma” and “scatterplot3d” packages to conduct principal component analysis (PCA) showed that these lncRNAs could be reliably used to construct signatures.

### Functional enrichment analysis

To understand the functions of DEGs, we used the R package “limma” to identify differentially expressed genes between high-risk and low-risk groups. Then, functional enrichment analysis of differentially expressed genes was performed using Gene Ontology (GO) and Kyoto Encyclopedia of Genes and Genomes (KEGG) databases.

### Immune-related function analysis and TMB analysis

To assess immune status in low-risk and high-risk groups, we used BiocManager “limma”, BiocManager “GSVA”, BiocManager “ABGSEase”, “phearmap”, and “reshape2” packages to draw the heatmap of KIRP immune-related functions. Draw waterfall plots using the BiocManager “maftools” package. In addition, we also compared TMB between high-risk and low-risk groups and plotted TMB survival curves. The difference between TMB and patient survival was determined, and a *p*-value < 0.05 was considered statistically significant.

### High mutant genes and KIRP tumor immunoassay

We used the TCGA database-immunoassay (https://www.aclbi.com/static/index.html#/immunoasy) and the TIMER algorithm to observe the distribution of KIRP immune scores in tumor tissues and normal tissues. Afterward, we analyzed the expression distribution of KIRP immune checkpoint genes in tumor tissues and normal tissues. Then, we investigated the relationship between six genes with high mutation frequency (TTN, MET, KMT2C, PKHD1, SETD2, and KMT2D) and KIRP immune infiltration. Finally, to further investigate the association between highly mutated genes and KIRP tumor immunity, we correlated the KIRP immune checkpoint genes (CTLA4, HAVCR2, PDCD1, PDCD1LG2, and TIGIT) with high mutation frequency genes (TTN, MET, KMT2C, PKHD1, SETD2, and KMT2D).

### Drug sensitivity analysis and screening of potential KIRP drugs

By (http://bioinfo.life.hust.edu.cn/GSCA/#/drug), we analyzed the correlation between drug sensitivity of high mutant genes and their expression in the cancer therapeutic response portal (CTRP) database. It provides some basis for the mechanism of drug treatment. Finally, using the “ggpubr” package screened potential drugs for KIRP patients.

## Results

### Identification of cuproptosis-related lncRNAs and building prognosis signature

**FIGURE 1:**
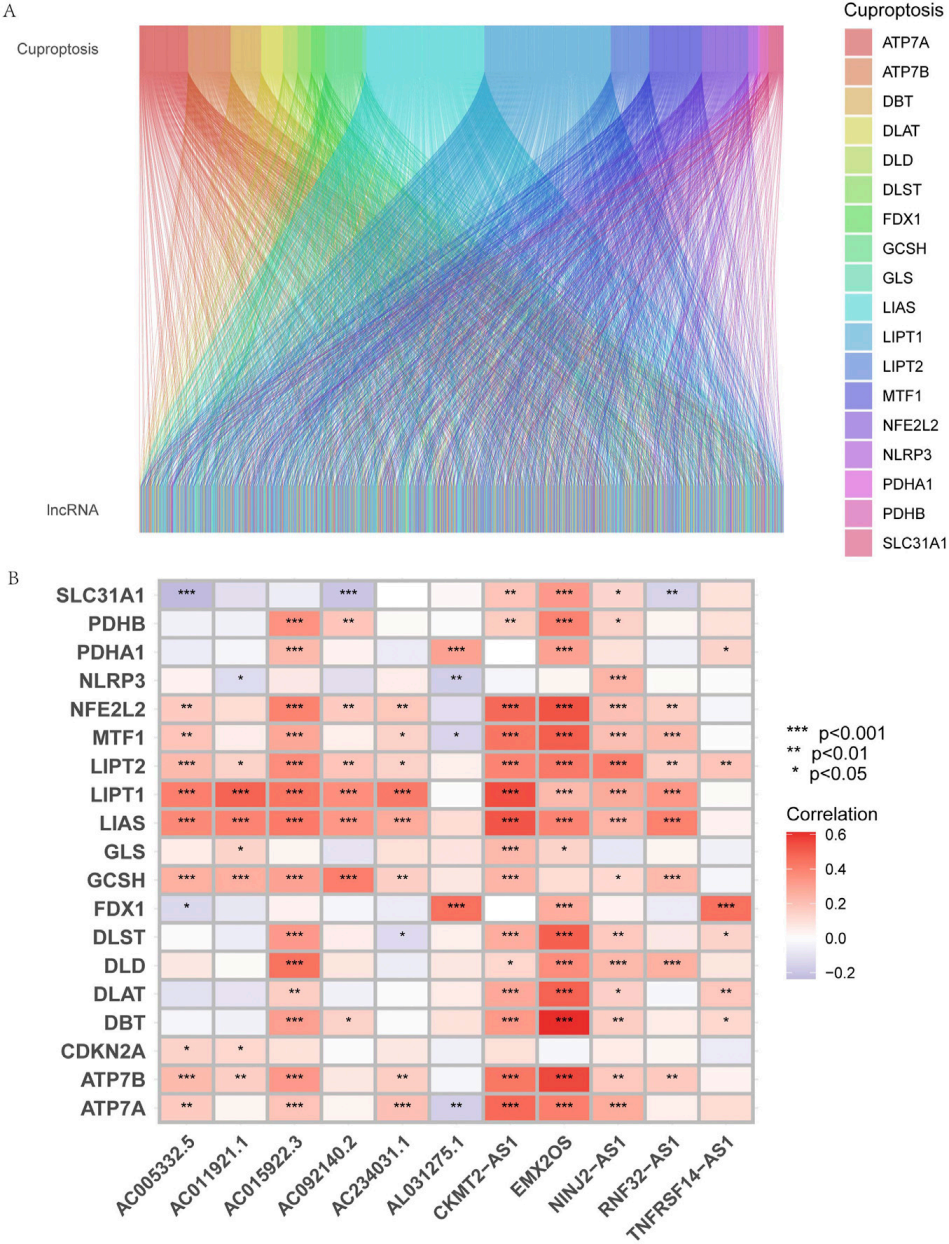
Identification of cuproptosis-related lncRNAs and construction of prognosis signatures. **(A)** Sankey diagram showed the results of cuproptosis-related genes and cuproptosis-related lncRNAs co-expression. **(B)** The correlation heatmap showed the relationship between cuproptosis-related lncRNA signatures and cuproptosis-related genes. Red represents positive correlations, and blue represents negative correlations.

**FIGURE 2:**
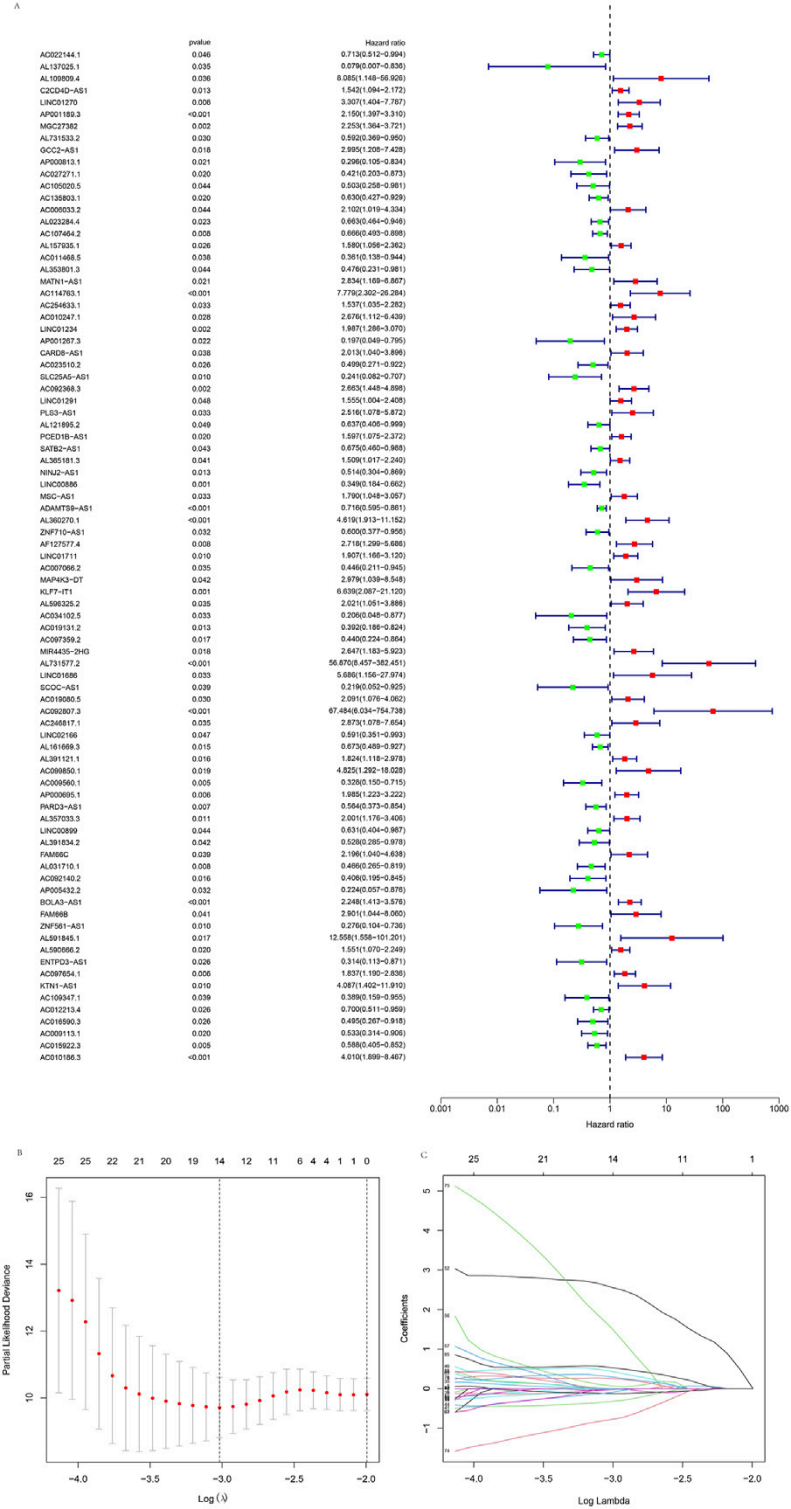
Identification of the cuproptosis-related lncRNAs. **(A)** The forest plot showed different lncRNAs for high and low risk, with red representing high-risk lncRNAs and green representing low-risk lncRNAs. **(B)** Lasso regression screened cuproptosis-related lncRNAs at the minimum point of cross-validation. **(C)** The trajectory of each independent variable.

Using | R | > 0.4 and *p* < 0.001 as analysis criteria, we extracted 19 cuproptosis-related genes and 16,876 lncRNAs from the KIRP cohort of the TCGA database for co-expression analysis and identified 3,203 cuproptosis-related lncRNAs. Using a Sanky plot, we visualized co-expression relationships between cuproptosis-related lncRNAs and cuproptosis-related genes (Figure 1A). The heatmap showed the association between lncRNAs and cuproptosis-related genes (Figure 1B). In the training group, cuproptosis-related lncRNAs were identified by Lasso regression analysis, 85 cuproptosis-related lncRNAs were identified by univariate Cox regression analysis (Figure 2A), and 11 cuproptosis-related lncRNAs were identified as independent prognosis factors by multivariate Cox analysis. Then, basing the expression of 11 lncRNAs calculated the risk scores of each sample (Figures 2A–C). Risk score = AC234031.1* 2.07217627199546 + TNFRSF14-AS1* (-0.823489225724154) + … + AC015922.3 (-1.32818876134062) (Supplementary Table S3).

### Survival analysis of the signature

**FIGURE 3:**
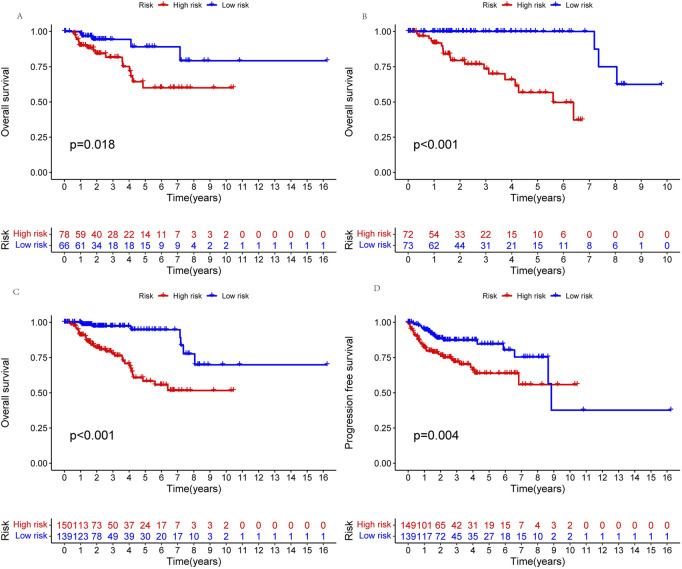
Kaplan-Meier survival analyses of patients. Patients were divided into high-risk and low-risk groups based on the median risk score to predict overall survival (OS) and progression-free survival (PFS) in each subgroup. **(A)** OS in the testing group. **(B)** OS in the training group. **(C)** OS in all groups. **(D)** PFS in all groups.

**FIGURE 4:**
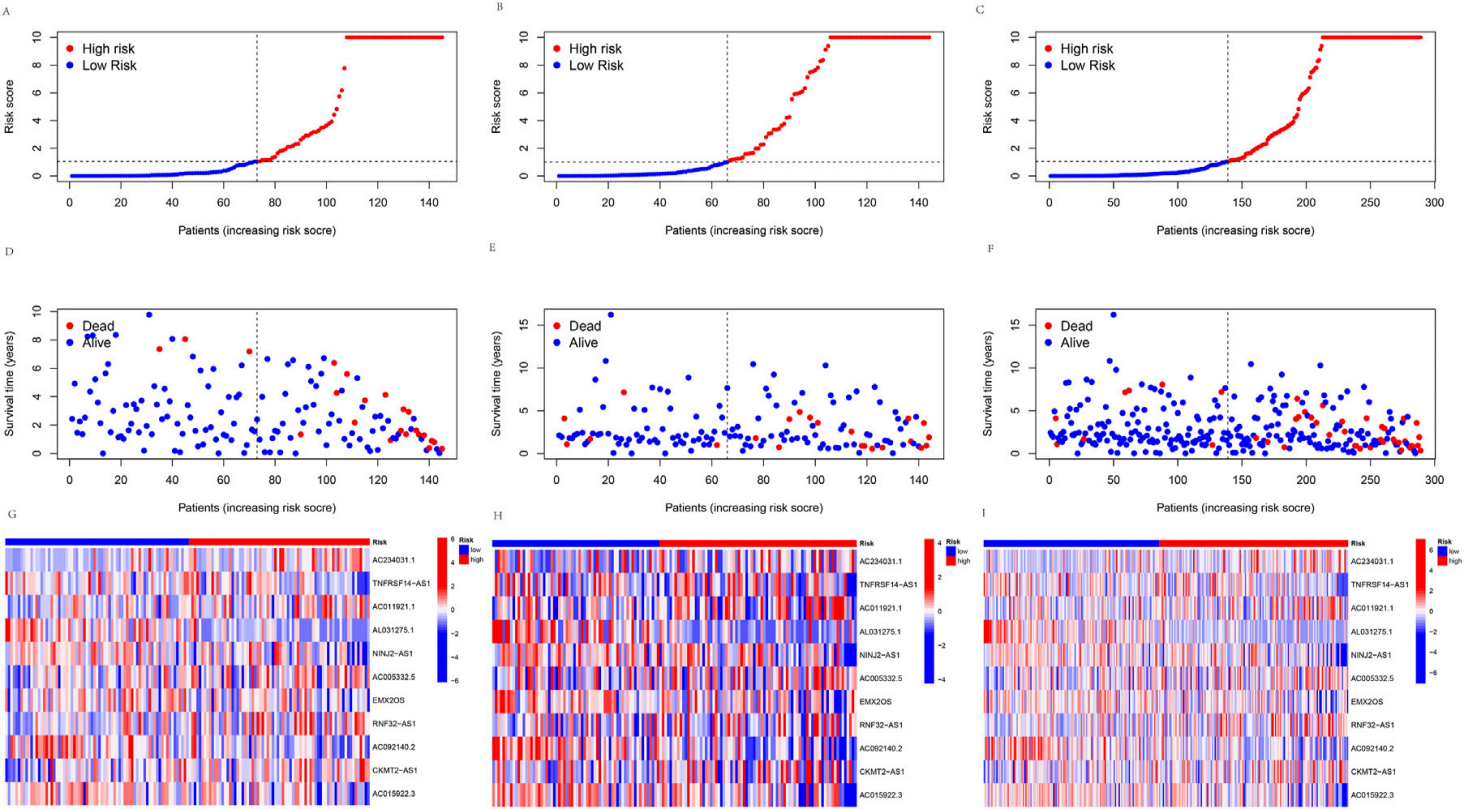
Predicting the performance of signature. The risk curve is based on the risk score for each sample in the **(A)** training group, **(B)** testing group, and **(C)** all groups, where red and blue dots indicate high- and low-risk samples, respectively. The scatter plot is based on the survival status of each sample from **(D)** the training group, **(E)** the testing group, and **(F)** all groups, where red and blue dots indicate death and survival, respectively. The heatmap represented the signature of lncRNAs in the **(G)** training group, **(H)** testing group, and **(I)** all groups.

A prognosis model was constructed using multiple Cox regression results to investigate further the prognosis ability of 11 cuproptosis-related lncRNAs in KIRP. We proceeded risk score for each patient and divided the KIRP sample into high-risk and low-risk groups based on the median risk score = 1. We found that OS and PFS were significantly higher in the low-risk group than in the high-risk group in training, testing, and all groups (Figures 3A–D). The risk curve reflects the relationship between different risk groups and survival status in KIRP patients, and we found that low-risk patients had lower mortality than high-risk patients (Figures 4A–F). High-risk and low-risk levels for 11 lncRNAs are shown in the heatmap; For example, AC234031.1, AC011921.1, AC005332.5, RNF32-AS1, and CKMT2-AS1 are high-risk lncRNAs and TNFRSF14-AS1, AL031275.1, NINJ2−AS1, EMX2OS, AC092140.2, and AC015922.3 are low-risk lncRNAs (Figures 4G–I).

### Independent analysis of the prognosis factor

**FIGURE 5:**
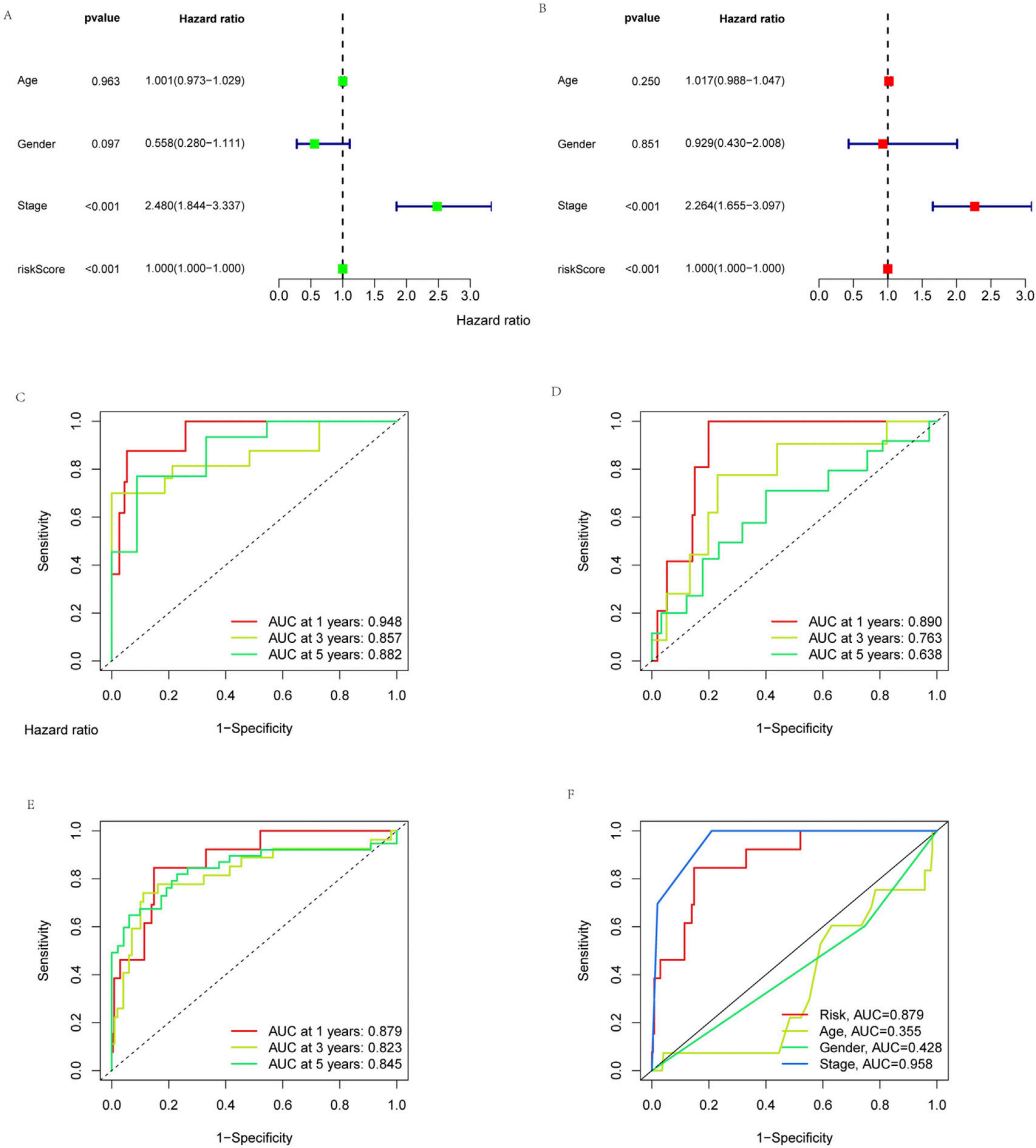
Independent analysis of prognosis factor. The prognosis value of the signature for KIRP. **(A)** Univariate and **(B)** multivariate independent prognosis analysis to analyze whether the risk score was independently associated with OS. 1-, 3-, and 5-year area under the ROC curve (AUC) of signature in the **(C)** training, **(D)** testing, and **(E)** all groups. **(F)** ROC curves for the risk score (AUC = 0.879) and other clinical features.

To determine whether risk signatures are likely to be independent prognosis factors, univariate and multivariate Cox regression analyses were used to investigate the prognosis value of cuproptosis-related lncRNA signatures in KIRP. Multivariate Cox regression results showed that the risk score (hazard ratio = 1.000, 1.000-1.000; *p* < 0.05) and stage (hazard ratio = 2.480, 1.844-3.337; *p* < 0.05) of cuproptosis-related lncRNA signatures were significantly associated with patient’s OS (Figures 5A, B). The result showed that the risk signature is an independent prognosis factor in KIRP. In addition, the AUCs for stage and risk score were better than gender and age in ROC curves, which further illustrates the reliability of the risk model (Figure 5F). Similarly, In the training group, the AUCs for 1-year, 3-year, and 5-year OS were 0.948, 0.857, and 0.882, respectively (Figure 5C); in the testing group, the AUCs for the 1-year, 3-year, and 5-year OS were 0.890, 0.763, and 0.638, respectively (Figure 5D). In all groups, the AUCs for 1-year, 3-year, and 5-year OS were 0.879, 0.823, and 0.845, respectively (Figure 5E). The above results indicate that this prognosis signature has reliable diagnostic significance.

### Building nomogram and PCA

**FIGURE 6:**
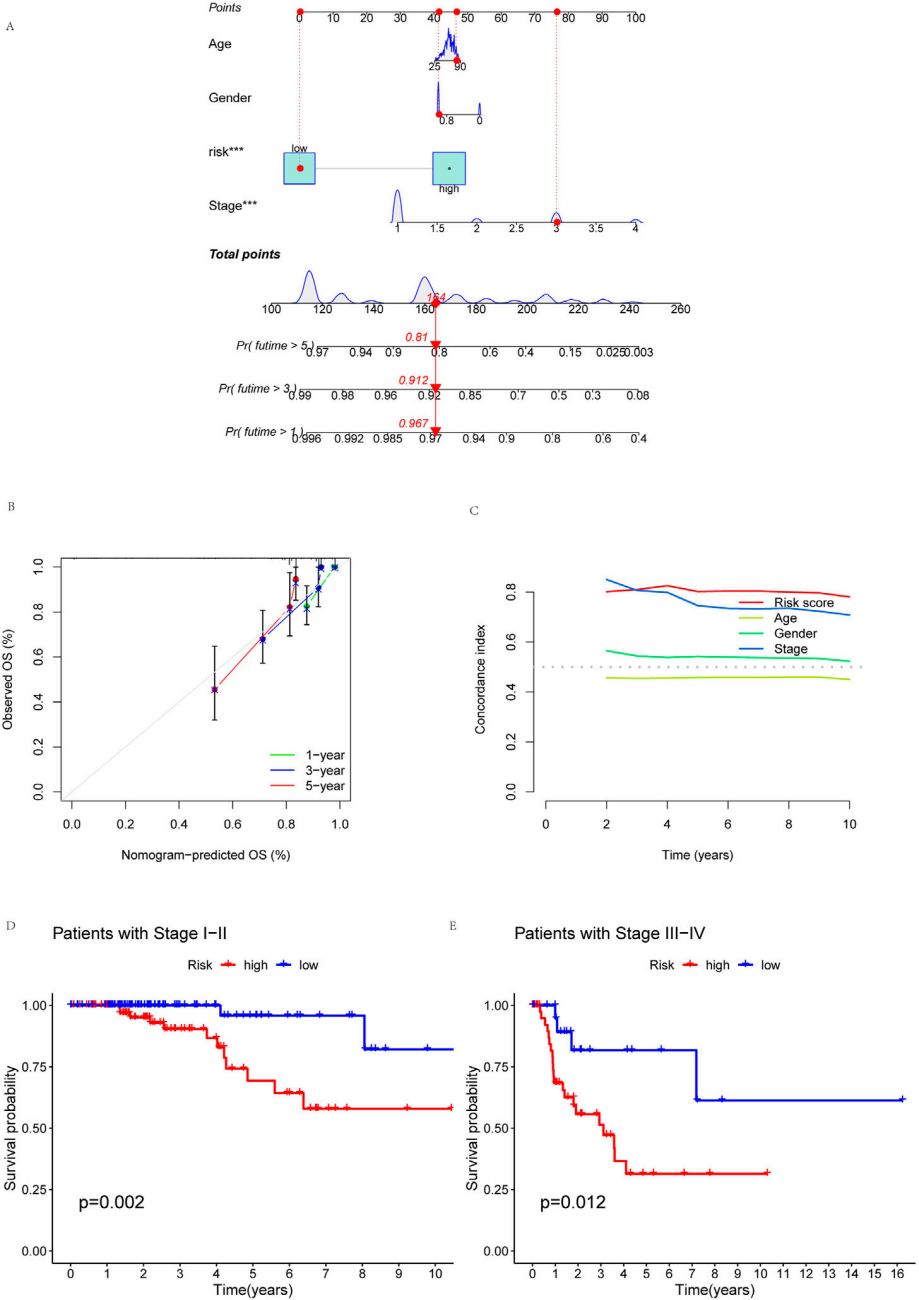
Building nomogram. Nomogram and clinical subgroups for predicting KIRP outcomes. **(A)** Nomogram to predict the OS in KIRP. **(B)** Calibration curves for 1, 3, and 5 years. **(C)** C-index curve analyzed the concordance index of the risk score. Patients were grouped to see if the model applied to KIRP patients at **(D)** stages I-II and **(E)** III-IV.

**FIGURE 7:**
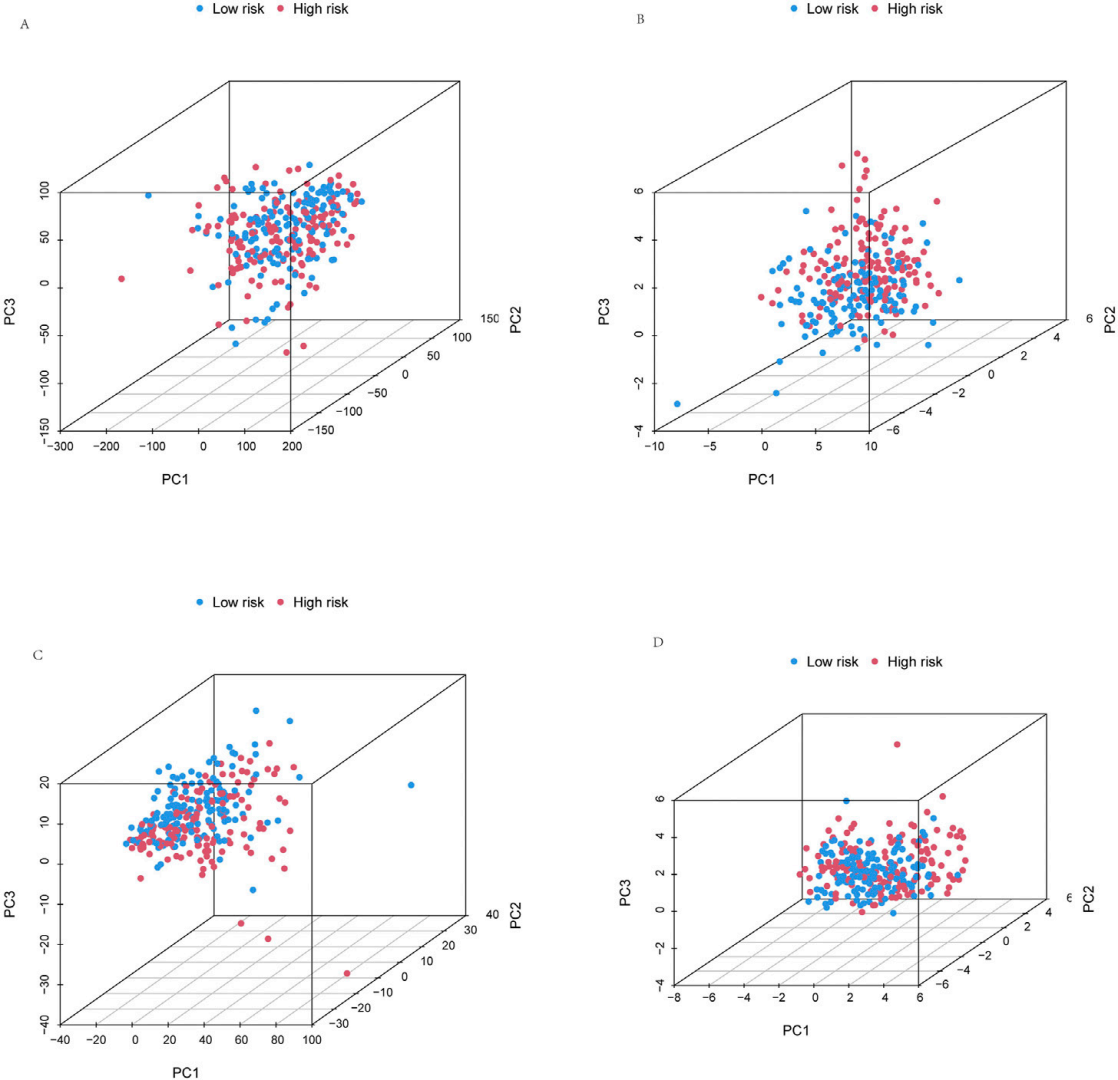
Principal component analysis. PCA observed the distribution of patients according to **(A)** all genes. **(B)** Cuproptosis-related genes. **(C)** Cuproptosis-related lncRNAs. **(D)** Risk lncRNAs. Patients with high-risk scores are denoted in red, while those with low-risk scores are represented in blue.

To more reliably predict OS at 1, 3, and 5 years in patients with KIRP, we developed a nomogram that combined clinicopathological features and risk scores. The patient’s combined risk score was 164, suggesting that the predicted survival probability of this patient in the following 1, 3, and 5 years was 96.7%, 91.2%, and 81.0% (Figure 6A). Calibration curves of OS at 1-, 3-, and 5 years affirmed the predictive power of the prognosis model (Figure 6B). In addition, the risk score’s C-index value was also higher than age, gender and stage (Figure 6C). To validate the clinical grouping model, we divided the patients into early (I-II) and late (III-IV) groups by stage. The results showed that there was a significant difference in OS between the early-stage and late-stage patients (*p* < 0.05) (Figures 6D, E), which indicated the predictive reliability. Finally, we performed PCA. PCA results showed that risk lncRNAs (Figure 7D) could better classify KIRP patients into low and high-risk groups compared to all genes (Figure 7A), cuproptosis-related genes (Figure 7B), and cuproptosis-related lncRNAs (Figure 7C), this suggests that these lncRNAs can be used more reliably to construct prognosis signatures.

### Functional enrichment analysis

**FIGURE 8:**
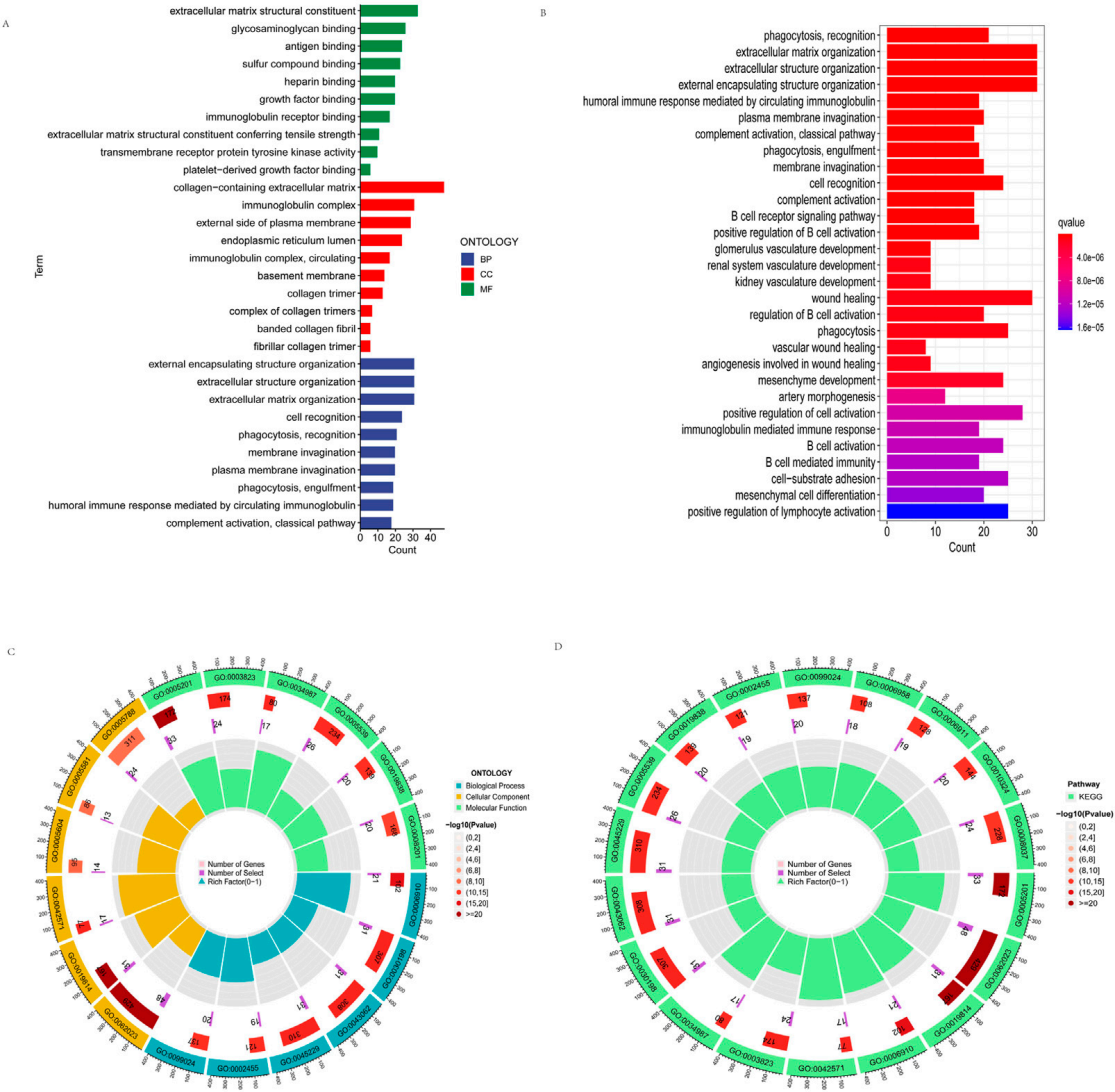
Functional enrichment analysis. **(A)** GO enrichment analyses of the differentially expressed genes. **(B)** KEGG enrichment analyses of the differentially expressed genes. **(C,D)** shows the location, the number of genes, and the number and proportion of differential genes of GO and KEGG.

To elucidate the function of DEGs, we utilized GO and KEGG databases to analyze related pathways. We found that GO is divided into three categories: biological pathway (BP), cytological component (CC), and molecular function (MF). These DEGs were mainly involved in membrane invagination, phagocytosis, recognition, cell recognition, fibrillar collagen trimer, banded collagen fibril, basement membrane, circulating, immunoglobulin complex, transmembrane receptor protein tyrosine kinase activity, immunoglobulin receptor binding, antigen binding in GO analysis (Figure 8A). In addition, KEGG pathway analysis showed that DEGs are mainly involved in cell activation, positive regulation of B cell activation, B cell receptor signaling pathway, kidney development, regulation of B cell activation, B cell-mediated immunity B cell vasculature activation, immunoglobulin mediated immune response, renal system recognition development, glomerulus development (Figure 8B). Figure 8 C, D shows the location, the number of genes, and the number and proportion of differential genes for GO and KEGG.

### Immune-related function analysis and TMB analysis

**FIGURE 9:**
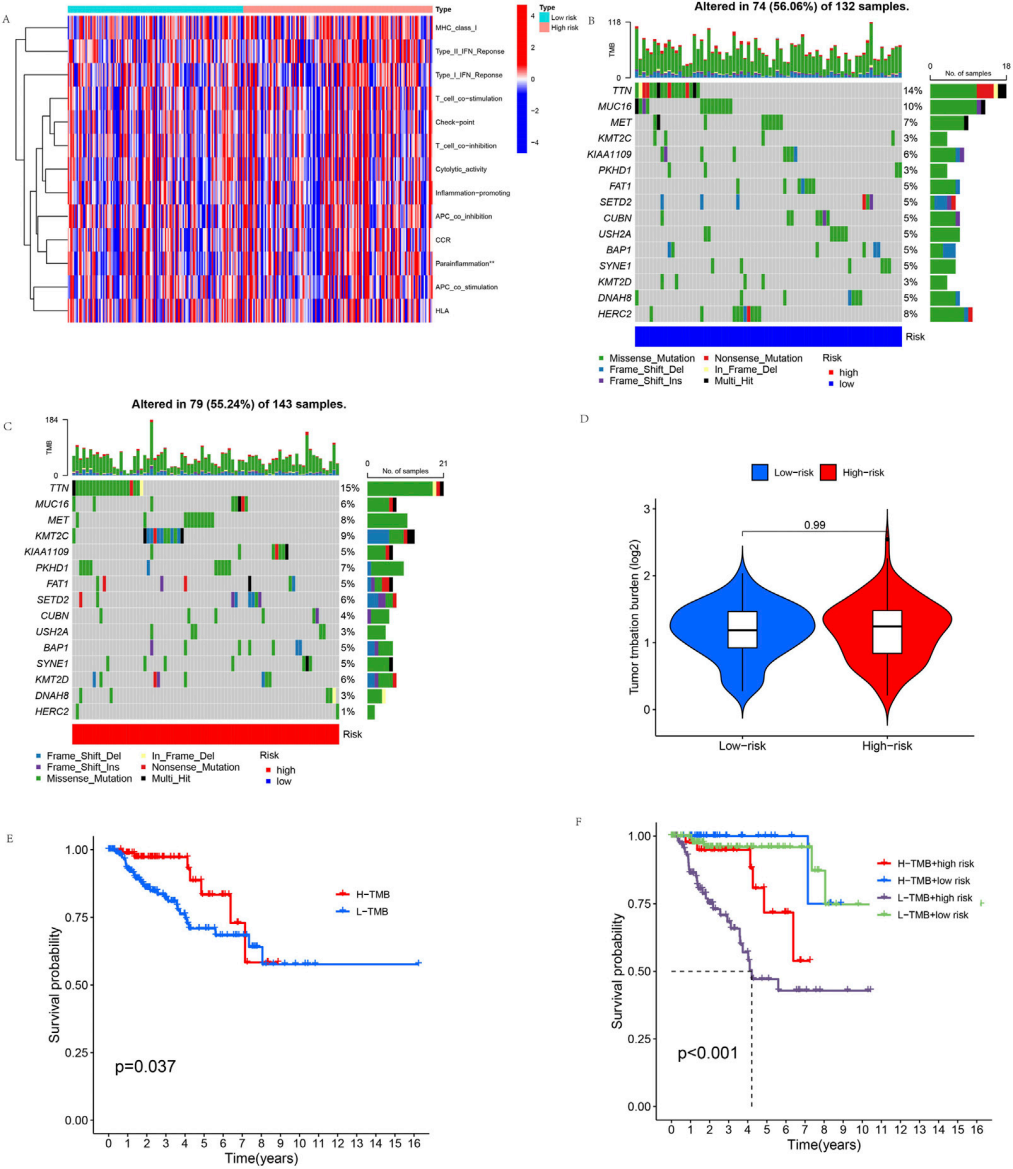
Immune-related function analysis and TMB. **(A)** Immune-related functions of the 11 cuproptosis-related lncRNAs. **(B,C)** These waterfall plots show somatic mutations of the most significant 15 genes among **(B)** low-risk and **(C)** high-risk KIRP patients. **(D)** Differential TMB in high-risk and low-risk groups in KIRP. **(E) **Survival curves for the high-TMB and low-TMB groups in KIRP. **(F)** The combined TMB-risk survival curve. (*p < 0.05, **p < 0.01,***p < 0.001, asterisks (*) stand for significance levels).

We evaluated the immune status of the high-risk and low-risk groups by immune-related function analysis. The results showed a significant difference in parainflammation response between the high-risk and low-risk groups (*p* < 0.05), and parainflammation responses were significantly higher in the high-risk group than in the low-risk group. But there was no significant difference in other immune functions (Figure 9A). In addition, the mutation frequencies of TTN, MET, KMT2C, PKHD1, SETD2, and KMT2D genes were higher in the high-risk group than in the low-risk group, but the mutation frequencies of MUC16, KIAA109, CUBN, USH2A, DNAH8, and HERC2 genes were significantly lower than in the low-risk group (Figures 9B, C). We compared tumor gene mutation frequencies and further investigated the difference in TMB between different risk groups. The result showed no statistically significant TMB between the two groups (Figure 9D). We then explored the survival analysis of TMB, and the results showed that OS was significantly better in the high-TMB group than in the low-TMB group (Figure 9E). Finally, we performed a combined TMB-risk analysis, and survival showed a clear difference between them (Figure 9F).

### High mutant genes and KIRP tumor immunoassay

**FIGURE 10:**
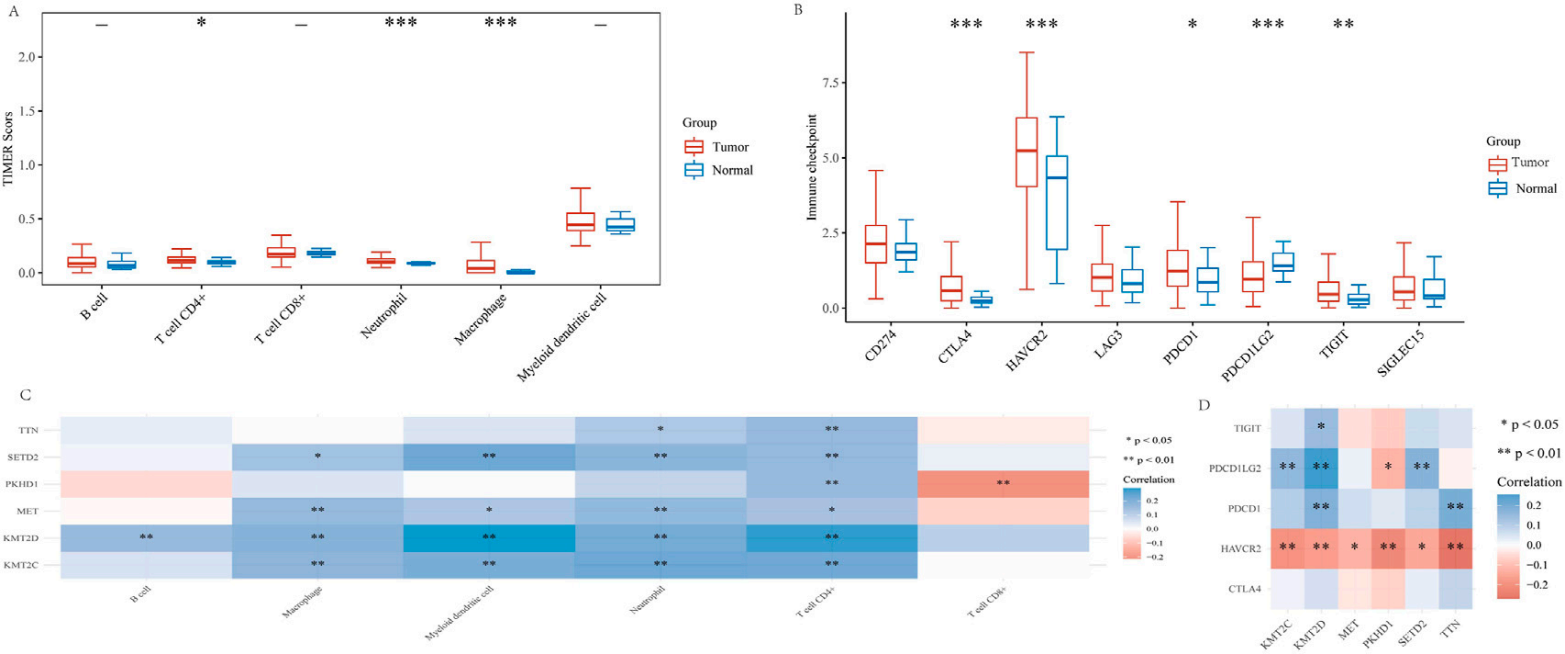
High mutant genes and KIRP tumor immunoassay. **(A)** In patients with KIRP, the TIMER algorithm was used to observe differences in immune cells in tumor versus normal tissue. **(B)** Different expressions of immune checkpoints in tumor tissue and normal tissue in KIRP (*p < 0.05, **p < 0.01, ***p < 0.001, asterisks (*) stand for significance levels. **(C)** The correlation between six high-mutation genes and immune infiltration. **(D)** The correlation analysis between six high-mutation genes and immune checkpoints. (*p < 0.05, **p < 0.01, ***p < 0.001, asterisks (*) stand for significance levels).

Because cuproptosis plays a crucial role in developing the tumor immune microenvironment in KIRP, we used the TIMER algorithm to observe the distribution of KIRP immune scores in tumor tissues and normal tissues. The results showed that the expression of CD4 + T cells, neutrophils and macrophages in tumor tissues was significantly higher than in normal tissues (Figure 10A). Afterward, we analyzed the expression distribution of KIRP immune checkpoint genes in tumor tissues and normal tissues. The results showed that CTLA4, HAVCR2, PDCD1, PDCD1LG2, and TIGIT significantly differed in tumor tissues and normal tissues (Figure 10B). In addition, we analyzed the association of six genes (TTN, MET, KMT2C, PKHD1, SETD2, KMT2D) with KIRP immune infiltration. The results showed that TTN, MET, KMT2C, PKHD1, SETD2 and KMT2D expression were positively correlated with CD4 + T cell expression, and their correlations were statistically significant. Only PKHD1 expression was negatively correlated with CD8 + T cell expression (Figure 10C). To further investigate the relationship between KIRP tumor immunity, we performed the correlation analysis between KIRP immune checkpoint genes (CTLA4, HAVCR2, PDCD1, PDCD1LG2, and TIGIT) and high mutation frequency genes (TTN, MET, KMT2C, PKHD1, SETD2, and KMT2D). We found that HAVCR2 expression was negatively correlated with TTN, MET, KMT2C, PKHD1, SETD2, and KMT2D expression, PDCD1 expression was positively correlated with TTN and KMT2D expression, PDCD1LG2 expression was positively correlated with KMT2C, KMT2D, and SETD2 expression, TIGIT expression was positively correlated with KMT2D expression, and their correlation was statistically significant (Figure 10D).

### Drug sensitivity analysis and screening of potential KIRP drugs

**FIGURE11:**
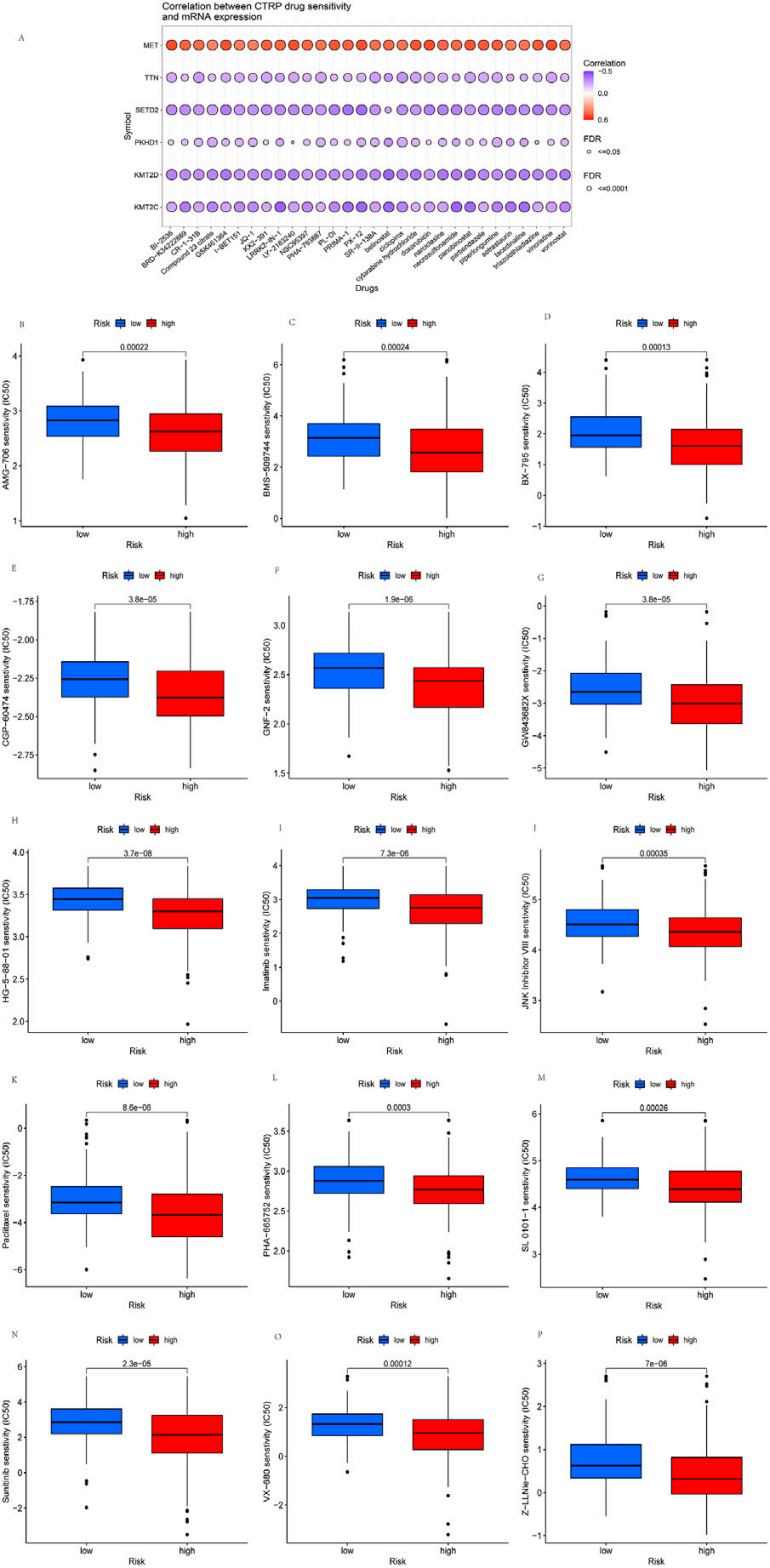
Drug sensitivity analysis and screening of potential KIRP drugs. **(A)** Drug-sensitivity analysis of six high-mutation genes in KIRP. **(B–P)** Potential 15 drugs for KIRP.

Drug therapy is an essential means of KIRP tumor treatment through drug sensitivity analysis to provide a specific basis for the mechanism of drug treatment. Drug sensitivity analysis was performed to explore the potential of TTN, MET, KMT2C, PKHD1, SETD2, and KMT2D as targets for KIRP drug scanning. The results showed that TTN, KMT2C, PKHD1, SETD2, and KMT2D expression were negatively correlated with most drugs in the CTRP. In contrast, the expression of MET was positively correlated with most drugs in the CTRP (Figure 11A). Afterward, we screened 15 potential drugs that differ between high-risk and low-risk patients (Figure 11B–P), such as AMG-706, BMS-509744, BX-795, CGP-60474, GNF-2, GW843682X, HG-5-88-01, Imatinib, JNK Inhibitor VIII, Paclitaxel, PHA-665752, SL 0101-1, VX-680, Z-LLNle-CHO, Sunitinib. We found that the concentration inhibiting cell growth by 50% (IC50) was significantly lower in high-risk patients than in low-risk patients, representing high-risk patients as more sensitive to these drugs.

## Discussion

Our study identifies 11 cuproptosis-related lncRNAs, constructs their prognosis-related signatures, and finds AC234031.1, AC011921.1, AC005332.5, RNF32-AS1 and CKMT2-AS1 are high-risk lncRNAs, while TNFRSF14-AS1, AL031275.1, NINJ2-AS1, EMX2OS, AC092140.2, and AC015922.3 are low-risk lncRNAs. Relevant studies have shown that the lncRNA signature of AC005332.5 is a potential diagnostic biomarker for HBV-related hepatocellular carcinoma (HCC) patients (Fu et al., 2022), the lncRNA signature of CKMT2-AS1 is closely related to the prevention of colorectal cancer (Zhuang et al., 2022), the lncRNA signature of TNFRSF14−AS1 can be used as a prognosis marker for bladder cancer (Wang et al., 2021a), the lncRNA signature of AC015922.3 is a novel biomarker for esophageal squamous cell carcinoma (Liu et al., 2020), and downregulation of lncRNA EMX2OS can independently predict shorter recurrence-free survival in classical papillary thyroid cancer (Gu et al., 2018). However, this study first investigated the prognosis profile of NINJ2-AS1, RNF32-AS1, AC234031.1, AC011921.1, AC092140.2, and AL031275.1. According to the results of functional enrichment analysis, we found that DEGs were highly enriched in B cell receptor signaling pathway, kidney development, regulation of B cell activation, B cell-mediated immune B cell vascular activation, transmembrane receptor protein tyrosine kinase activity, renal system recognition development, glomerulus development, etc. Therefore, we can reasonably assume that cuproptosis may be closely related to the development and immunotherapy of KIRP.

In immune-related function analysis, only parainflammation responses were differences in the immune-related function. Parainflammation is a “parainfluenza” state that exists between basal homeostatic conditions and actual inflammation and is an adaptive response of the immune system to low levels of tissue stress (i.e., low levels of “dangerous” stimuli), and its role is to maintain physiological balance in the body (Chen and Xu, 2015). Related studies have shown that parainflammation may be a driver of *p*53 mutagenesis significantly associated with the development and progression of cancer types containing *p*53 mutations (Aran et al., 2016). Similarly, Wang et al. found that microbiota-driven parainflammation is a factor leading to the carcinogenesis of colonic epithelial cells (Wang et al., 2021b). These provide a direction for future studies of KIRP therapeutic targets.

In TMB analysis, we found increased TTN expression in patients of the high-risk group. OS was significantly higher in the high TMB group than in the low TMB group. It has been previously demonstrated that TMB is an independent prognosis factor and can predict survival after immunotherapy for cancer types (Samstein et al., 2019). TTN is associated with prognosis in a variety of cancers (Zheng et al., 2021). Zhu et al. found that TTN can promote the proliferation and migration of prostate cancer by inhibiting miR-1271 levels, indicating that TTN may be a prognosis target for prostate cancer (Zhu et al., 2021). Cui et al. found that TTN could inhibit the proliferation and invasion of colorectal cancer cells by blocking the activation of PI3K/Akt/mTOR signaling by interacting with miR-497 (Cui et al., 2019). Similarly, Qi and Li (2020) found that TTN promoted the increase and migration of non-small cell lung cancer (NSCLC) by regulating the miR-491-5p/ZNF503 axis. Our results are consistent with previous findings and suggest that TTN may serve as a Prognosis therapeutic target in cancer.

In KIRP tumor immunoassays, CD4 + T Cell, neutrophil, and macrophage expression were significantly higher in tumor tissues than in normal tissues. CD4 + T cells are vital regulatory cells in the immune response and play an essential role in tumor development and progression. It has been found that CD4 + T cells infiltrating breast tumor tissue can effectively predict the survival of breast cancer and can examine patients through their signatures (Gu-Trantien et al., 2013). Neutrophils can produce and release active cytokines, such as IL6-1, IL-6 and vascular endothelial growth factor (VEGF), which alter the balance of inflammation and anti-inflammation in the tumor microenvironment, making inflammatory response biomarkers promising prognosis factors in renal cell cancer (RCC) (Fox et al., 2013). Related studies have demonstrated that neutrophils promote the occurrence of breast cancer and are closely associated with the therapeutic effect of breast cancer (Zhang et al., 2020). Similarly, Zhang et al. found that tumors with higher neutrophil-to-lymphocyte ratios in KIRP were larger and more advanced stages (Zhang et al., 2021a). To some extent, these related studies can affirm our study results.

In the correlation of KIRP immune infiltration, we found that only PKHD1 expression was negatively correlated with CD8 + T cell expression, and the correlation between the two was statistically significant. CD8 + T cell is closely related to the development of KIRP (Zhang et al., 2021b). CD8 +T cell is less expressed in recurrent renal cells but increases cancer-associated fibroblast (CAFs) infiltration compared to KIRP (Peng et al., 2022). PKHD1 gene is an essential factor leading to autosomal recessive polycystic kidney disease (ARPKD) in children. High PKHD1 mutations, on the other hand, may increase susceptibility to colorectal cancer (Ward et al., 2011). And related studies have shown that PKHD1 is associated with renal damage (Burgmaier et al., 2021).

In gene correlation analysis, we found that HAVCR2 expression was negatively correlated with TTN, MET, KMT2C, PKHD1, SETD2 and KMT2D expression. HAVCR2 is also a valuable gene in the KIRP immune checkpoint. Previous studies have shown a significant association between HAVCR2 methylation and mRNA expression and immune cell infiltration in melanoma (Holderried et al., 2019). Liu et al. (2018) found that high expression of HAVCR2 in hepatocellular carcinoma indicates poor prognosis, and HAVCR2 could also enhance Treg-mediated immunosuppression by mediating effector T-cell depletion and apoptosis. Cheng et al. (2015) also found that HAVCR2 can be used as an independent prognostic factor to predict the prognosis of gastric cancer patients in gastric cancer. Similarly, our study also showed that HAVCR was highly expressed in tumors and correlated with the development and immunotherapy of KIRP.

The results of the drug sensitivity analysis showed that MET expression was positively correlated with most drugs in the CTRP. Albiges et al. (2014) found that high MET expression is present in all KIRPs and is a therapeutic target. MET is an effective drug target, and the outcome of KIRPs is generally worse when treated with conventional therapies (Pal et al., 2021). We screened 15 potential drugs that differed between high-risk and low-risk patients. HG-5-88-01 and GNF-2, Z-LLNle-CHO are the most sensitive in high-risk patients. Currently, there is a lack of drug studies with HG-5-88-0. GNF-2 inhibits the growth of Bcr-abl positive cells, improves survival in chronic myelogenous leukemia, and can potentially treat solid tumors (Jones and Thompson, 2020). Meng et al. (2011) found that GSI-I (Z-LLNle-CHO0) triggered cell death in precursor B acute lymphoblastic leukemia by inhibiting γ-secretase and proteasome. As can be seen, the therapeutic range of these drugs is small, and we should increase the study of these drugs in KIRP in the future. *In vitro*, sunitinib inhibited cell proliferation by targeting the cytosolic MEK/ERK and SAPK/JNK pathways in RET/PTC1 cells. And sunitinib also has good activity against anaplastic thyroid cancer (ATC) cells *in vivo* (Ferrari et al., 2019). Zhang et al. (2013) found that autophagy inhibition enhanced paclitaxel’s preferential toxicity on folliculin-deficient renal carcinoma cells. These related studies largely corroborate the therapeutic value of the 15 potential drugs selected for this study in cancer. It can provide direction for future studies of drug therapy in KIRP patients.

This study provides insights into cuproptosis-related lncRNA signatures and their functional and immune correlation. However, our study is still limited. First, we did not perform a tumor immunity correlation analysis for lowly mutated genes. Second, *in vivo* and *in vitro* experiments should be performed to confirm our results further. Again, our study data are based on public databases and lack useful information on new clinical patients.

## Conclusion

Our study identified 11 cuproptosis-related lncRNAs and their prognosis signatures in KIRP. We have also validated that prognosis models can reliably predict the prognosis of KIRP patients. We preliminarily elaborated on the function of cuproptosis-related lncRNAs. The immune-related functional analysis assessed immune status in high and low-risk groups and found differences only in parainflammation responses. We investigated the difference in TMB between different risk groups and its association with survival and found that OS was better in the high-TBM group. Finally, we performed a drug sensitivity analysis, screening 15 potential drugs, and found that patients in the high-risk group were highly sensitive to potential drugs. Our study can provide some direction for subsequent investigation of the therapeutic target or prognosis value of cuproptosis-related lncRNAs in KIRP.

## Data Availability

The original contributions presented in the study are included in the article/Supplementary Material, further inquiries can be directed to the corresponding author.
